# Phenotypic and Genotypic Characterization of Clinical Isolates Belonging to the *Acinetobacter calcoaceticus-Acinetobacter baumannii* (ACB) Complex Isolated From Animals Treated at a Veterinary Hospital in Switzerland

**DOI:** 10.3389/fvets.2019.00017

**Published:** 2019-02-05

**Authors:** Sabrina Püntener-Simmen, Katrin Zurfluh, Sarah Schmitt, Roger Stephan, Magdalena Nüesch-Inderbinen

**Affiliations:** ^1^Institute for Food Safety and Hygiene, Vetsuisse Faculty, University of Zurich, Zurich, Switzerland; ^2^Institute of Veterinary Bacteriology, Vetsuisse Faculty, University of Zurich, Zurich, Switzerland

**Keywords:** *Acinetobacter*, *bla*_OXA−51_-like, genotypes, antimicrobial resistance, animals

## Abstract

**Objectives:** We investigated a collection of strains belonging to the *Acinetobacter calcoaceticus*-*Acinetobacter baumannii* (ACB) complex obtained from a veterinary clinic with regard to their genetic relatedness, presence of antibiotic resistance genes and antimicrobial susceptibility profiles.

**Methods:** Fifty-eight ACB-complex strains from animals treated at a veterinary clinic between 2006 and 2017, and seven strains collected from the hospital environment during 2012 were analyzed. Assignment to sequence types (ST) and international complexes (IC) was done by multilocus sequence typing (MLST) according to the Pasteur scheme. Genes encoding carbapenemases, aminoglycoside-modifying enzymes, macrolide-, quinolone- and co-trimoxazole resistance genes, the IS*Aba1* element, virulence associated *intI1* genes and plasmid associated toxin-antitoxin markers were identified by microarray. Genes encoding *bla*_OXA−51_-like carbapenemases were amplified by PCR and sequenced. Susceptibility profiles were determined by disc diffusion or by broth microdilution.

**Results:** Among 50 *A. baumannii* isolates from animals, two predominant clones were observed linked to CC1 (*n* = 27/54% of the isolates) and CC25 (*n* = 14/28%), respectively. Strains of IC I harbored *bla*_OXA−69_, *aac(3*′*)-la, aadA1, sul1, intI1*, and *splA/T* genes. Isolates belonging to CC25 possessed *bla*_OXA−64_. Six (12%) isolates belonging to CC2 and carrying *bla*_OXA−66_ were also noted. One isolate belonged to CC10 (*bla*_OXA−68_), one to CC149 (*bla*_OXA−104_), the remaining isolate was assigned to ST1220 and possessed *bla*_OXA−116_. Of six environmental *A. baumannii*, four (66.7%) belonged to CC25 (*bla*_OXA−64_), one (16.7%) to CC2 (*bla*_OXA−66_) and one to CC3 (*bla*_OXA−71_). Nine isolates (eight from animals and one environmental strain) were non-*baumannii* strains and did not harbor *bla*_OXA−51_-like genes. None of the isolates carried *bla*_OXA−23_, *bla*_OXA−48_, or *bla*_OXA−58_, and none were resistant to carbapenems.

**Conclusions:** Clonal lineages of the veterinary *A. baumannii* isolates in our collection are identical to those globally emerging in humans but do not harbor *bla*_OXA−23_. *A. baumannii* CC25 may be specific for this particular veterinary clinic environment.

## Introduction

The genus *Acinetobacter* is ubiquitous in diverse environments and as of today comprises 60 validly published species names (www.szu.cz/anemec/Classification.pdf). A list of *Acinetobacter* spp. also available at http://www.bacterio.net (1). In clinical settings, the species belonging to the *Acinetobacter calcoaceticus*- *Acinetobacter baumannii* (ACB) complex are of greatest importance (2). The ACB complex currently *comprises Acinetobacter baumannii* and its close relatives, *A. calcoaceticus, A. dijkshoorniae* (3), *A. lactucae* (4), *A. nosocomialis, A. pittii*, (5), and *A. seifertii* (6). Currently, *A. dijkshoorniae* and *A. lactucae* are considered conspecific (7). Hence, there exist to date six distinct ACB complex species with formal nomenclatural recognition.

*A. baumannii* is the most frequent *Acinetobacter* species isolated from patients in intensive care units (ICUs) and is the causative agent of ventilator associated pneumonia, catheter-related bloodstream infections, meningitis, and wound infection, often causing clonal outbreaks involving critically ill patients (8, 9). By constrast, *A. calcoaceticus*, although found sometimes in clinical specimens, seems to be more environmental, has unknown clinical significance and is usually well-susceptible to antibiotics (10).

The diversity of strains in epidemiology studies of *A. baumannii* is frequently investigated by multilocus sequence typing (MLST) using either the Oxford or the Pasteur scheme (11, 12). The majority of outbreak strains reported globally belong to IC I, IC II, and IC III, corresponding to clonal complexes (CC)1, CC2, and CC3 of the Pasteur scheme (9, 12–14).

Treatment of infections is frequently compromised due to the fact that ACB complex strains possess multiple intrinsic and acquired mechanisms that may result in antimicrobial resistance (10, 15). Overexpression of intrinsic ß-lactamases and of multidrug resistance efflux pumps, loss of outer membrane proteins and mutations in the quinolone resistance-determining regions (QRDRs) of the *gyrA* and *parC* are commonly detected in ACB isolates (10). Hence, following CLSI guidelines, ACB isolates are considered intrinsically resistant to antibiotics such as aminopenicillins, aztreonam, ertapenem, trimethoprim, chloramphenicol, macrolides, and fosfomycin (16).

Importantly, in addition to the presence of chromosomally located *bla*_OXA−51_-like genes encoding for naturally occurring carbapenemases, plasmid mediated carbapenem resistance genes including *bla*_OXA−23_, *bla*_OXA−48_, and *bla*_OXA−58_ have emerged globally in *A. baumannii* further restricting therapeutic options for treating infections in humans, with *bla*_OXA−23_ harboring *A. baumannii* representing one of the most problematic hospital-acquired human pathogens (17).

Data on molecular characteristics and antimicrobial resistance mechanisms of *Acinetobacter* of veterinary origin are still scarce compared to those of isolates from humans. However, it has been shown that *A. baumannii* isolated from animals may share clonal lineages and possess identical transmissible antibiotic resistance genes to those from humans, suggesting common pathways and/or sources of infection (15, 18). Furthermore, reports on the emergence of infections due to carbapenem resistant *A. baumannii* in hospitalized companion animals are of concern and emphasize the need for epidemiological studies and surveillance in order to maintain veterinary and public health (19–22).

The present study was designed to characterize clinical isolates belonging to the ACB complex originating from companion animals and horses hospitalized during 2006–2017 at a university veterinary clinic in Switzerland by (i) determining the genetic relatedness using multilocus sequence typing, (ii) performing genetic profiling using a microarray-based assay, and (iii) assessing their antimicrobial susceptibility profiles.

## Materials and Methods

### Bacterial Isolates

Between 2006 and 2017, a total of 93 non-duplicate *Acinetobacter* spp. isolated from hospitalized animals (one strain per animal) were obtained. Only isolates with clinical significance were collected. In addition, strains taken from the hospital environment during 2012 (*n* = 7) were included in the study. Strains were identified to the level of the genus *Acinetobacter* using the VITEK® 2 Compact system (Biomérieux, Nürtingen, Germany).

Species identification was performed by matrix-assisted laser desorption/ionization time-of- flight mass spectrometry (MALDI-TOF–MS, Bruker Daltronics, Bremen, Germany) and by amplification and sequencing of the 350 bp highly variable zone 1 of the *rpoB* gene (23, 24). Custom sequencing was done by Microsynth, Balgach, Switzerland.

In total, 65 ACB complex strains were identified, including 56 *A. baumannii* (50 isolates from animals, and six environmental strains), seven *A. pittii* (six animal, and one environmental isolate), and two animal *A. calcoaceticus* isolates.

The 58 animal strains originated from horses (*n* = 35), cats (*n* = 7), dogs (*n* = 6), chicken (*n* = 3), rabbits (*n* = 2), Andean bear (*n* = 1), cattle (*n* = 1), donkey (*n* = 1) reptile (*n* = 1), and rodent (*n* = 1) admitted to the veterinary clinic of the University of Zürich, Switzerland. The isolates were cultured from wounds (*n* = 20), abscesses (*n* = 19), urine (*n* = 4), synovial fluid aspirations (*n* = 2), tracheobronchial secretions (*n* = 2), abdominal aspiration (*n* = 1), alveolus (*n* = 1), bladder wall (*n* = 1), eye swab (*n* = 1), implant (*n* = 1), pus (*n* = 1), surgical sites (3), and other sites (*n* = 2).

In addition, strains collected from the hospital environment during 2012 (*n* = 7) were included in the study.

Non-ACB-complex strains (*n* = 35) comprising 21 *A. lwoffii/*‘*A. pseudolwoffii*’ (25), three *A. guillouiae*, three *A. radioresistens, two A. beijerinckii*, two *A. towneri, one A. gandensis, one A. junii*, one *A. parvus*, and one *A. ursingii* were not included in this study.

In accordance with local legislation, ethics approval was not required and no animal experiments were carried out for this study.

### Multilocus Sequence Typing

Multilocus sequence typing was performed according to the scheme developed by the Pasteur Institute (12). This scheme involves PCR amplification and sequencing of internal fragments of seven housekeeping genes (*fusA, gltA, pyrG, recA, cpn60, rpoB*, and *rplB*). Primers and PCR conditions are listed at the *A. baumannii* MLST database website http://pubmlst.org/abaumannii/. Sequencing of the amplification products was performed by Microsynth (Balgach, Switzerland). Sequences were uploaded to http://pubmlst.org/abaumannii/ to identify alleles and sequence types. The population structure of STs of the *A. baumannii* isolates was evaluated using the goeBURST software (http://www.phyloviz.net/goeburst/). CCs were defined as single-locus (SLVs) and double-locus variants (DLVs).

### Identification of Antimicrobial Resistance Genotypes

DNA was purified using the DNeasy Blood & Tissue Kit (Qiagen, Hilden, Germany), according to manufacturer's protocol.

Isolates were genotyped using the oligonucleotide based microarray CarbDetect AS-2 Kit (Alere Technologies GmbH, Jena, Germany) to detect all currently known relevant carbapenemase genes, extended-spectrum ß-lactamase (ESBL) genes, aminoglycoside, macrolide, quinolone and co-trimoxazole resistance genes found in Enterobacteriaceae and Pseudomonadales (26). Additional markers included the IS*Aba1* element, integrase and transposase genes, and plasmid associated toxin-antitoxin (T/A) markers. An overview of the target genes and multiplex labeling, hybridization and data analysis has been described by Braun et al. (26). In brief, DNA was labeled internally with biotin-11-dUTP using a linear amplification protocol to generate single stranded (ss) DNA. Biotin labeled ssDNA was transferred and hybridized with DNA probes in oligonucleotide microarray strips. Hybridization was detected using streptavidin-horseradish peroxidase and a dye precipitation. The signals were detected using the platform ArrayMate Reader provided by Alere Technologies GmbH.

PCR and DNA sequencing analyses of *bla*_OXA−51_-like genes in *A. baumannii* isolates was carried out using custom synthesized primers (Microsynth, Balgach, Switzerland) and conditions published previously (27). Nucleotide sequences were analyzed with the CLC Main Workbench 8.0.1 and the BLASTN program of NCBI (http://www.ncbi.nlm.nih.gov/blast/).

Screening for the plasmid-mediated colistin resistance genes *mcr-1, mcr-2, mcr-3, mcr-4*, and *mcr-5* was performed by PCR using custom synthesized primers (Microsynth, Balgach, Switzerland) and conditions described previously (28).

The *mcr-1* harboring strain OW3E1 (29) and plasmid “Plasmid-MCR2-Positivkontrolle” (P. Keller, personal communication) were used as positive controls.

### Phenotypical Characterization of Antibiotic Susceptibility

Antimicrobial susceptibility testing was carried out according to Clinical and Laboratory Standards Institute (CLSI) performance standards (16), using the disk-diffusion method and the antibiotics cefotaxime (CTX), cefepime (FEP), ciprofloxacin (CIP), sulfamethoxazole/trimethoprim (SXT), gentamicin (GM), and tetracycline (TE). Minimal inhibitory concentrations (MIC) of imipenem were determined using the E-Test® (bioMérieux, Marcy L'Etoile, France) according to manufacturer's protocol. Determination of the MIC of colistin was performed by broth microdilution according to the European Committee on Antimicrobial Susceptibility Testing EUCAST (eucast.org).

The degrees of antimicrobial resistance among the ACB complex isolates was defined in accordance to Falagas and Karageorgopoulos (30).

Multidrug resistance (MDR) was thereby defined as resistance to three or more classes of antimicrobial agents including β-lactams, fluoroquinolones, sulfonamides, aminoglycosides, tetracyclines, and polymyxins, and excluding those which cannot be regarded as potentially effective, i.e., to which ACB complex strains are intrinsically resistant: aminopenicillins, aztreonam, ertapenem, trimethoprim, chloramphenicol, macrolides and fosfomycin (16).

## Results

**Figure 1:**
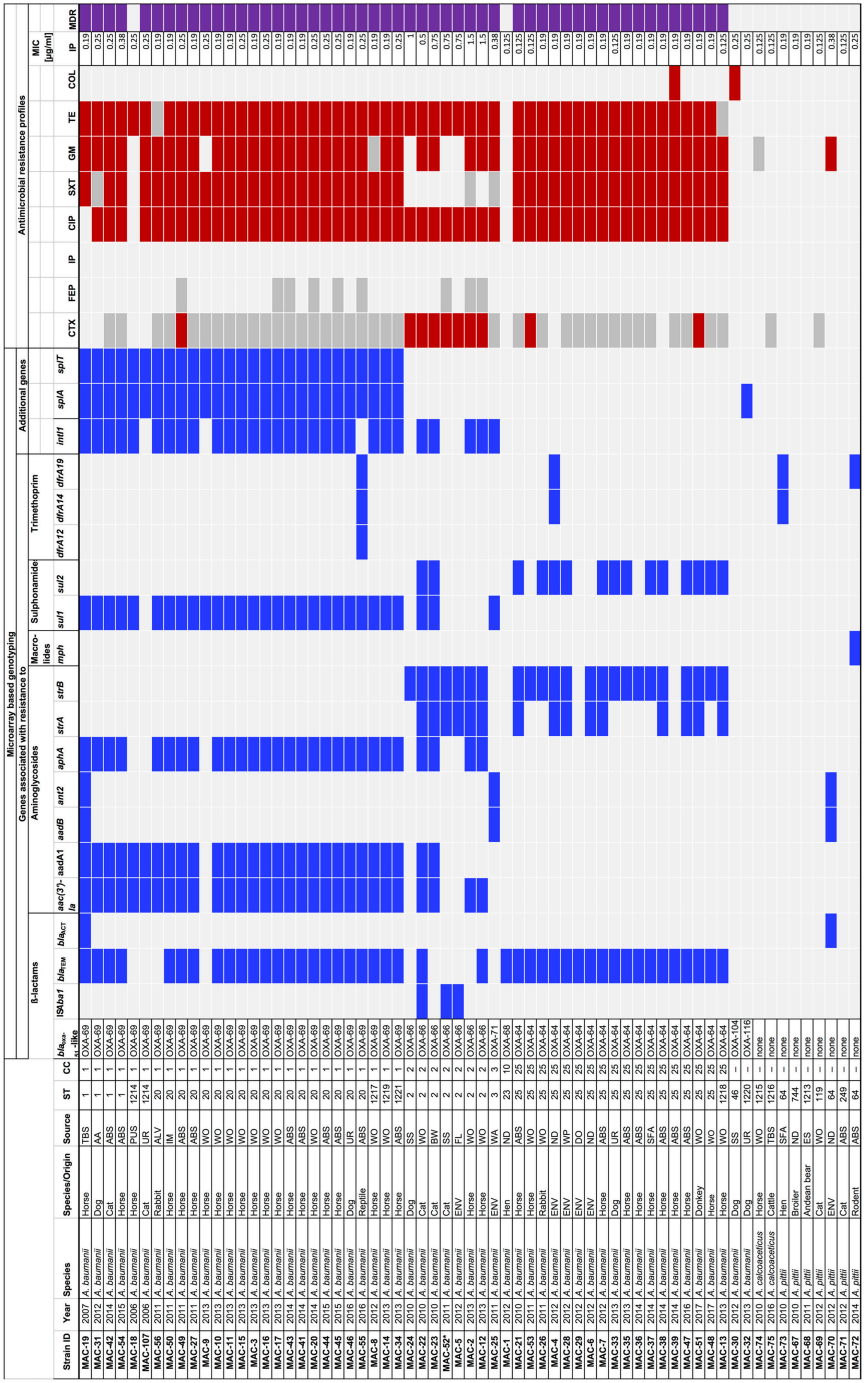
Molecular and phenotypic characterization of strains belonging to the *Acinetobacter calcoaceticus*-*Acinetobacter baumannii* (ACB) complex isolated from animals and the environment at a veterinary hospital in Switzerland, 2006–2017. AA, abdominal aspiration; ABS, abscess; ALV, alveolus; AZM, azithromycin; BW, bladder wall; CC, clonal complex; CIP, ciprofloxacin; COL, colistin; CTX, cefotaxime; DO, door; ES, eye swab; ENV, environment; FEP, cefepime; FL, floor; GM, gentamicin; IM, implant; IP, imipenem; MIC, minimal inhibitory concentration; MDR, multidrug resistant; SS, surgical site; SFA, synovial fluid aspiration; ST, sequence type; SXT, sulfamethoxazole/trimethoprim; TBS, tracheobronchial secretion; TE, tetracycline; UR, urine; WA, water; WP, water pipe; WO, wound. ^*^ In isolate MAC-52, the IS*Aba1* element was not associated with *bla*_OXA−66_. Blue squares, positive result; red squares, resistant to a specific antimicrobial; gray squares, intermediately resistant to a specific antimicrobial; light gray squares, negative result or susceptible to a specific antimicrobial; purple squares, multidrug resistant.

An overview of the isolates, their origins, molecular and phenotypic characteristics is shown in Figure 1.

Overall, 58 clinical isolates belonging to the ACB complex were collected from animals admitted to the veterinary hospital of Zürich, Switzerland between 2006 and 2017. The majority thereof (50/86.2%) were *A. baumannii* isolates collected from horses (*n* = 34), dogs (*n* = 6), cats (*n* = 5), rabbits (*n* = 2), and from one chicken, one donkey and one reptile, respectively. Six (10.3%) of the clinical isolates were *A. pittii* collected from cats (*n* = 2), chicken (*n* = 2), and from one Andean bear and one rodent, respectively. Two isolates (3.4%) were *A. calcoaceticus* from a cow and from a horse, respectively.

Environmental isolates collected from the premises of the veterinary hospital during 2012 included six *A. baumannii* and one *A. pittii* (Figure 1).

**Figure 2:**
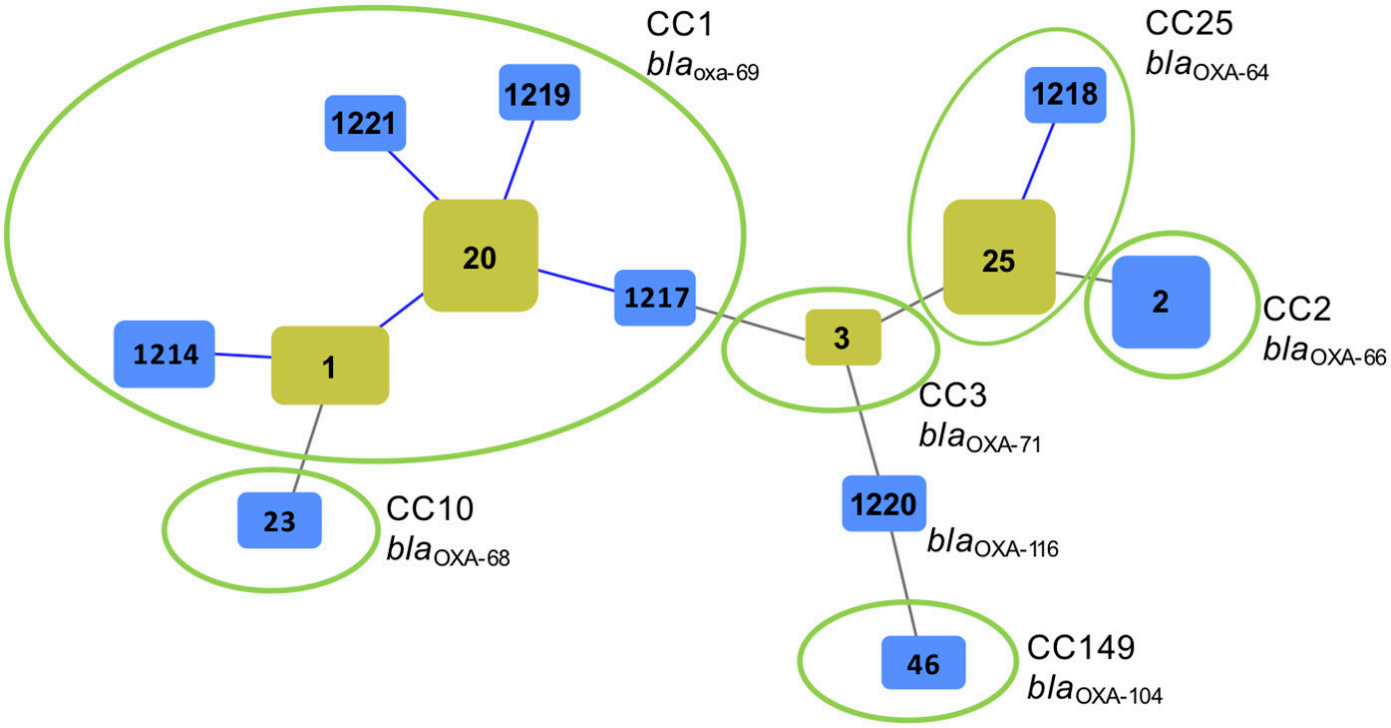
Genetic relatedness of *Acinetobacter baumannii* isolated from animals and the environment at a veterinary hospital in Switzerland during 2006–2017 using multilocus sequence typing (MLST) and goeBurst (Phylowiz). The sizes of the squares reflect the number of strains belonging to a particular sequence type (ST). Blue links show single locus variants (SLVs). Founder STs are highlighted in yellow. Green circles indicate isolates grouped into a clonal complex (CC) harboring a particular *bla*_OXA−51_-like allele.

Multilocus sequence typing revealed that the majority (27/48.2%) of the 56 *A. baumannii* isolates belonged to ST20 and its SLVs ST1, ST1217, and ST1219, as well as its DLV ST1214. eBurst analysis assigned these sequence types to clonal complex CC1 (Figure 2). Fourteen (25%) of the *A. baumannii* strains belonged to ST25 and its SLV ST1218 and were assigned to CC25. Of the remaining isolates, six (10.7%) belonged to ST2 (CC2), and one (1.8%) to ST23 (CC10) and one to ST46 (CC149). One isolate typed and ST1220 and was not assigned to any CC (Figure 2).

Microarray based genotyping revealed the presence of *bla*_OXA−51_-like genes in all *A. baumannii* isolates. None of the *A. pittii* or *A. calcoaceticus* isolates tested positive for *bla*_OXA−51_-like genes (Figure 1).

Sequencing analysis of the *bla*_OXA−51_-like genes revealed the presence of *bla*_OXA−69_ in all 27 *A. baumannii* belonging to CC1 (Figures 1, 2). The *bla*_OXA−64_ gene was detected in the 18 *A. baumannii* isolates belonging to CC25, and *bla*_OXA−66_ was identified in the seven strains belonging to CC2_._ Other alleles included *bla*_OXA−71_, *bla*_OXA−104_, and *bla*_OXA−116_ (Figures 1, 2).

The ß-lactamase genes *bla*_TEM_ and *bla*_ACT_ were found in 44 and 2 of the isolates, respectively (Figure 1).

Other *bla* genes encoding for carbapenemases (e.g., OXA-23, OXA-48, OXA-58, KPC, or NDM), or for acquired ESBLs (e.g., PER, VEB, or CTX-M types) were not detected.

The IS*Aba1* element which accounts for enhanced expression of *bla*_OXA−51_-like genes was detected adjacent to *bla*_OXA−66_ in two of the isolates (MAC5 and MAC22, Figure 1). In one isolate (MAC-52) the element was not associated with *bla*_OXA−66_ (Figure 1).

Genes associated with resistance to aminoglycosides, macrolides, sulphonamides and trimethoprim were detected as shown in Figure 1. The *aac(3*′*)-Ia, aadA1, aphA*, and *sul1* genes occurred predominantly in association with the presence of the class 1 integrase gene *intI1* in *A. baumannii* belonging to CC1 and CC2, whereas the majority of strains that lacked the *intI1* gene harbored *strA* and/or *strB* and *sul2* (Figure 1).

The type II T/A genes *splA* and *splT* were identified in all *A. baumannii* belonging to CC1.

In one strain (MAC-32), the toxin-antitoxin system was incomplete (Figure 1).

None of the isolates tested positive for *mcr* genes.

The antimicrobial susceptibility profiles of the isolates are summarized in Figure 1. The majority (52/92.9%) of the 56 *A. baumannii* strains was resistant to three or more classes of antimicrobials and were MDR according to Falagas and Karageorgopoulos (30). By contrast, among the *A. calcoaceticus* and *A. pittii* strains, one isolate (MAC-70) was resistant to gentamicin, the rest remained susceptible to all tested antimicrobials, and none were MDR.

Overall, 51 (91%) of the *A. baumannii* isolates were resistant to ciprofloxacin and to tetracycline, respectively, 47 (83.9%) were resistant to gentamicin, 43 (76.8%) were resistant to sulfamethoxazole-trimethoprim, and 10 (17.9%) were resistant to cefotaxime. Two (3.6%) isolates were resistant to colistin. All were susceptible to cefepime and imipenem, with MIC of imipenem ranging from 0.125 to 1.5 μg/ml (Figure 1).

## Discussion

There is growing concern that multidrug resistant, *bla*_OXA−23_ harboring *A. baumannii* in hospitalized companion animals and horses may be emerging as a threat to veterinary and public health (15). However, information on *A. baumannii* in veterinary medicine is still limited and there is a lack of comparable data to strains isolated from humans (15, 31). In this study, we provide a molecular and phenotypic analysis of strains belonging to the ACB complex isolated from diseased animals admitted to the veterinary hospital of the university of Zürich, Switzerland during 2006–2017. The main limitations of this study include its retrospective design and its restriction to a single center.

The two predominant lineages of *A. baumannii* comprised CC1, which is a globally distributed clade (9), and CC25, a lineage that has been responsible for epidemics in different European countries (32).

There are few reports on *A. baumannii* isolated from pets in Switzerland and overall, these isolates belonged primarily to CC1and CC2 (33, 34). Likewise, Ewers et al. (19) observed a prevalence of 26% of CC2 among *A. baumannii* recovered from animals hospitalized in various veterinary clinics in Germany, which is remarkably higher than the prevalence of 12% observed in this study. By contrast, *A. baumannii* ST25 (CC25) was not described in either of these studies. Its abundance in the collection of ACB isolates from the veterinary hospital of Zürich suggests that this clinical setting may be likely support the spread of this particular clonal lineage. Moreover, of the six environmental *A. baumannii* recovered during 2012, four (66.7%) belonged to ST25 (CC25), suggesting the existence of an environmental reservoir of this ST in or outside of the hospital setting. Its prevalence in the hospital environment may also be due to the elevated resistance to desiccation and high biofilm-forming capacity on abiotic surfaces, as demonstrated for this particular sequence type (35).

Notably, *A. baumannii* ST25 has recently been isolated from pets in France (20, 21). In both studies, the isolates possessed *bla*_OXA−23_ and were resistant to carbapenems, whereas the isolates analyzed in the current report possessed intrinsic *bla*_OXA−51_ –like carbapenemases only. Interestingly, the isolates from France were detected in companion animals in the community. Compared to clinical settings, little is known about *Acinetobacter* carriage in animals beyond these settings, but several studies during the last decade have detected A. baumannii in dogs in the community (21, 36), domestic birds (37), livestock (38) and other farmed animals such as mink (39). These reports indicate that community-acquired A. baumannii infections among animals may be increasing and that animals outside clinical settings may represent a reservoir for A. baumannii, including carbapenem resistant strains (15).

Overall, the *bla*_OXA−51_ alleles identified in the *A. baumannii* isolates correlated with their respective CCs, in accordance with previous observations for human isolates (27, 40).

Two isolates possessed the IS*Aba1* element upstream of *bla*_OXA−66_. As reported earlier, IS*Aba1* mediates overexpression of *bla*_OXA−51_-like enzymes, resulting in resistance to carbapenems (41, 42). However, there was no difference in the MICs of imipenem for these two isolates compared to those lacking the IS*Aba1* insertion, confirming recent observations that resistance to carbapenems is not guaranteed only by the presence of IS*Aba1*, but depends on its orientation upstream of the *bla* gene (43, 44).

Aminoglycoside resistance genes were distributed unevenly among the A. baumannii isolates. The occurrence of *aadA1* and *aphA* in association with intI1 among CC1, CC2, and CC3 is supportive of previous observations (45). By contrast, these genes were not prevalent among the CC25 isolates, among which *strA* and *strB* genes predominated. This may indicate that interclonal horizontal gene transfer plays a minor role in the dissemination of aminoglycoside resistance in the isolates analyzed in this study.

Of the two colistin resistant isolates, one belonged to CC25, which also occurs in humans. Colistin resistance in human *Acinetobacter* isolates is a source of great concern, although to date, there have been no reports of *mcr* positive *Acinetobacter* spp. (46).

In general, there was a good correlation between the presence of resistance genes detected by microarray and the phenotype of the isolates. There was however, a discrepancy between results of genotypic and phenotypic testing for three of isolates containing sulfonamide resistance genes and 4 isolates harboring aminoglycoside resistance genes, where the presence of resistance genes did not correspond with phenotypic resistance. Conversely, there was a lack of *bla*_ESBL_ genes such as *bla*_GES_, *bla*_PER_, or *bla*_VEB_ that would explain the phenotypic resistance to cefotaxime observed in 10 isolates (10). On the other hand, IS*Aba1* and IS*Aba125* governed hyperexpression of the chromosomal *bla*_ADC_ cephalosporinase also leads to resistance to 3rd generation cephalosporins in *A. baumannii* (10, 47). Thus, further investigations targeting the genetic environment of *bla*_ADC_ in the cefotaxime resistant isolates are warranted to explain their phenotype.

In this study, we observed the presence of type II T/A genes *splA* and *splT* in *A. baumannii* CC1. Type II T/A systems are usually plasmid encoded and mediate plasmid maintenance through the post-segregational killing of plasmid-free daughter cells (48). The *splA* and *splT* genes are so far unique to *A. baumannii* and are encoded on small, ca. ~10 kb plasmids of the Rep-3 superfamily (49, 50). There is a lack of knowledge regarding these plasmids, however, some harbor *bla*_OXA−24_/*bla*_OXA−40_ or *bla*_OXA−72_ and are prevalent among carbapenem-resistant human clinical IC II isolates in Eastern Europe (49). The significance of the *splA/T* carrying plasmids identified among the CC1 isolates in this study remains to be investigated.

Finally, non-*baumannii* ACB complex species accounted for 13.8% of the animal, and 14.3% of the environmental isolates. These isolates were distinguished from *A. baumannii* strains by the lack of *bla*_OXA−51_-like genes, the low prevalence of acquired antibiotic resistance genes and high rate of susceptibility to antimicrobial agents, in agreement with observations from human isolates (8, 51).

In conclusion, this study provides a molecular and phenotypic analysis of ACB complex isolates obtained from animals admitted to a veterinary hospital in Switzerland during 2006–2017. Using established methods applied to isolates of human origin enabled the identification of clonal lineages and resistance determinants that occur globally among human isolates, including CC1 and CC25 *A. baumannii*. As opposed to *A. baumannii* CC1, CC25 isolates have infrequently been described in companion animals, but were prevalent among the isolates in this study. Contrasting to frequently occurring human clinical isolates worldwide, the veterinary ACB complex isolates in this study did not possess any known acquired carbapenemase genes. However, since *A. baumannii*, including CC25 isolates are emerging as *bla*_OXA−23_ carrying veterinary isolates in other countries, increased surveillance and targeted measures to prevent the dissemination of ACB complex strains are warranted.

## Author Contributions

RS designed the study. KZ, SP-S, and SS carried out the microbiological and molecular biological tests. SP-S, KZ, and MN-I analyzed and interpreted the data. MN-I drafted the manuscript. All authors read and approved the final manuscript.

### Conflict of Interest Statement

The authors declare that the research was conducted in the absence of any commercial or financial relationships that could be construed as a potential conflict of interest.
